# Podocyte RhoGTPases: new therapeutic targets for nephrotic syndrome?

**DOI:** 10.12688/f1000research.20105.1

**Published:** 2019-11-04

**Authors:** Moin A. Saleem, Gavin I. Welsh

**Affiliations:** 1 Bristol Renal, Bristol Medical School, University of Bristol, Dorothy Hodgkin Building, Whitson Street, Bristol, BS1 3NY, UK

**Keywords:** Podocyte, RhoGTPases, Nephrotic Syndrome.

## Abstract

Podocytes, or glomerular epithelial cells, form the final layer in the glomerular capillary wall of the kidney. Along with the glomerular basement membrane and glomerular endothelial cells, they make up the glomerular filtration barrier which allows the passage of water and small molecules and, in healthy individuals, prevents the passage of albumin and other key proteins. The podocyte is a specialised and terminally differentiated cell with a specific cell morphology that is largely dependent on a highly dynamic underlying cytoskeletal network and that is essential for maintaining glomerular function and integrity in healthy kidneys. The RhoGTPases (RhoA, Rac1 and Cdc42), which act as molecular switches that regulate actin dynamics, are known to play a crucial role in maintaining the cytoskeletal and molecular integrity of the podocyte foot processes in a dynamic manner. Recently, novel protein interaction networks that regulate the RhoGTPases in the podocyte and that are altered by disease have been discovered. This review will discuss these networks and their potential as novel therapeutic targets in nephrotic syndrome. It will also discuss the evidence that they are direct targets for (a) steroids, the first-line agents for the treatment of nephrotic syndrome, and (b) certain kinase inhibitors used in cancer treatment, leading to nephrotoxicity.

## Introduction

The kidney is vital for the maintenance of water and electrolyte homeostasis and for the removal of waste metabolites from the blood while retaining or reabsorbing useful components. The filtration unit of the kidney, the glomerulus is composed of a bundle of capillaries, which are highly permeable to water yet can retain larger macromolecules while selectively allowing passage of solutes. This selectivity is achieved by the action of the glomerular filtration barrier, which comprises the glomerular basement membrane, glomerular endothelial cells and glomerular epithelial cells—or podocytes ^1^. Terminally differentiated epithelial cells, podocytes are critical in preventing protein passage across the filtration barrier. They have branching and interdigitating processes, and filtration takes place through slits between these processes. A critical component of the filtration barrier, the slit diaphragm is an ultra-thin zipper-like structure that bridges the gap between interdigitating podocyte foot processes. It is a cell junction and signalling complex that is essential for regulating podocyte cytoskeletal dynamics ^2^.

Podocytes have a remarkably elaborate and highly specialised morphology that critically depends on an underlying network of dynamic and interconnected actin and microtubule polymers which allow them to respond rapidly to environmental changes to maintain a healthy filtration barrier ^3, 4^. Damage to this unique cytoskeletal architecture is a hallmark of glomerular disease, and the importance of correct regulation of this process can be demonstrated by the fact that a number of human nephrotic syndromes (NS) are caused by genetic mutations coding for podocyte-specific proteins or proteins that seem only dysfunctional within the podocyte and that are associated with the slit diaphragm or directly link it to the podocyte actin cytoskeleton ^5^. Therefore, an understanding of how the cellular architecture of the podocyte is regulated in health and how this is disrupted in disease is essential. Recent work, which will be discussed in this review, has highlighted the crucial role of the RhoGTPases—which act as molecular switches regulating actin dynamics—in the unique biology of the podocyte and the pathogenesis of glomerular diseases. Excitingly, recent work has also suggested that pathways that regulate RhoGTPases in the podocyte are the site of action for steroids, the main first-line treatment for NS, thus identifying novel biological pathways that can be targeted therapeutically ^6– 8^.

The RhoGTPases RhoA, Rac1 and Cdc42 act as molecular switches—cycling between an active GTP-bound form and an inactive GDP-bound form—that are crucial regulators of several cellular processes, including actin and microtubule cytoskeletal dynamics, cell morphogenesis and cell migration ^9^. The activity of these proteins is tightly regulated by a variety of guanine nucleotide exchange factors (GEFs), GTPase-activating proteins (GAPs) and guanine nucleotide dissociation inhibitors (GDIs) which act to control the ratio of the GTP- and GDP-bound forms ^9^. There is substantial evidence, reviewed previously ^10^, that the RhoGTPases play a crucial role in the regulation of podocyte biology whereby RhoA activation increases in actin stress fibres promoting a contractile phenotype whilst Rac1/Cdc42 activation increases lamellipodia/filopodia formation promoting cell motility ^11^. It is known that there is crosstalk between the GTPases and that tight regulation of their activities is crucial for maintaining a healthy podocyte phenotype as changes to the balance of the activity of the small GTPases lead to hypo- or hyper-motility of podocytes resulting in proteinuria ^7^. This was recently demonstrated in drosophila nephrocytes, which are structurally and functionally homologous to podocytes, where a tight balance of Rac1 and Cdc42 activity is essential to maintain the specialised architecture and function of this cell ^12^. The RhoGTPases appear to have different roles in specific disease states, and controversy remains as to the exact role that alterations in their activity play in disease pathogenesis ^11^. For example, alterations in Rac1 activity have been shown to be both beneficial and detrimental to the podocyte and this may depend on the type of podocyte injury involved ^11^. Inducible Rac1 activation specifically in podocytes in mice induces foot process effacement, proteinuria and the spectrum of NS ranging from minimal change disease to focal segmental glomerulosclerosis (FSGS) ^13, 14^. In contrast, Rac1 has also been shown to be important for glomerular repair as podocyte-specific knockout resulted in increased glomerulosclerosis via suppression of mammalian target of rapamycin (mTOR) activity in injured podocytes ^15^.

The RhoGTPases are known to regulate or are regulated by a number of factors implicated in NS, such as TRPC5, TRPC6 and suPAR ^16– 19^. Mutation analysis of patients with NS has recently revealed novel functional networks which regulate the GTPases and which are altered in disease-causing changes to the activity of these proteins. Braun *et al*. reported disease-causing mutations in genes encoding the nuclear pore complex proteins NUP107, NUP85 and NUP133 in patients with steroid-resistant NS and demonstrated that knockdown of any of these three proteins in podocytes led to the activation of Cdc42 ^20^. Ashraf *et al*. identified mutations in six different genes ( *MAGI2*, *TNS2*, *DCL1*, *CDK20*, *ITSN1* and *ITNS2*) which result in partially treatment-sensitive NS ^7^. These proteins interact and form complexes which are involved in the regulation of the RhoGTPases, especially RhoA and Cdc42 ^7^. MAGI2, TNS2, DCL1 and CDK20 form complexes that regulate RhoA whilst ITSN1 and 2 are GEFs for Cdc42. MAGI2 also forms a complex with the Rap1 GEF, RapGEF2, regulating Rap1 and this complex is lost in the presence of the MAGI2 mutations ^21^. Importantly, glucocorticoids, which are the standard treatment for NS in children, have been shown to act directly act on the podocyte ^22^ and a potential mechanism for their beneficial effect has been shown to be via these RhoGTPase regulatory complexes ^7^. In agreement with these findings that the mode of action of steroids may be via direct action on the podocyte to regulate the RhoGTPases, McCaffrey *et al*. have demonstrated that dexamethasone reduces the podocyte activity of Rac1, thereby increasing barrier function ^8^. However, modelling MAGI2 mutations in zebrafish suggest that the effectiveness of steroids in treating alterations in RhoGTPase activity depends on the specific genetic mutation involved and so unravelling the specific action and targets of these agents will be crucially important ^23^. Maeda *et al*. recently delineated another protein pathway regulating RhoGTPase activity in the podocyte, centred on Ca ^2+^/calmodulin-dependent kinase 4 (CaMK4)/synaptopodin, as a potential target for treating podocytopathies such as NS ^24^. Prevention of the degradation of the actin organising protein synaptopodin and subsequent stabilisation of the RhoA/Cdc42 signalling pathway by the direct action of the anti-proteinuric drug cyclosporin A on the podocyte have been reported ^25^. It has since been shown that increased expression of CaMK4, which is seen in FSGS, both alters the expression of Rac1 and RhoA and leads to the phosphorylation of the scaffold protein 14-3-3. Phosphorylation of 14-3-3 results in the proteolytic cleavage of the actin organising protein synaptopodin because of a loss of interaction between the two proteins, causing enhanced Rac1 signalling, decreased RhoA activity and increased podocyte motility ^24, 26^. A CaMK4 inhibitor ameliorated synaptopodin degradation, alterations in RhoGTPase activity and changes to motility in human podocytes. Furthermore, podocyte-specific targeting of this inhibitor prevented and reversed podocyte injury and renal disease in both the adriamycin mouse model of FSGS and mice exposed to lipopolysaccharide-induced podocyte injury ^24^. These results suggest that targeting the pathways regulating or regulated by the GTPase may be a novel therapeutic area for glomerular disease. Conversely, recent data suggested that kinase inhibitors such as deasatinib, used in clinical oncology, may have nephrotoxic effects by affecting RhoGTPase signalling in the podocyte ^27^. Therefore, a clear delineation of the complex network of protein interactions centred on the RhoGTPases in the podocyte and how they alter the specialised biology of this cell is becoming increasingly important.

In addition to the pathways already detailed, several other proteins have been identified to be important regulators of the GTPases in podocytes. For example, ARHGAP24 regulates Rho/Rac signalling balance in podocytes and mutations in this protein are associated with familial FSGS ^28^. Mutations in ARHGDIA (a GDI for Cdc42 and Rac1), KANK2, ARHGEF17 and FAT1 all result in altered RhoGTPase activity and cause NS ^29– 33^. SLIT-ROBO pGTPase-activating protein 2a (SRGAP2a) suppresses podocyte motility through inactivating RhoA and Cdc42 and is downregulated in patients with kidney disease ^34^. Trio, a GEF for Rac1, is expressed in podocytes and is significantly upregulated in glomeruli of patients with FSGS ^35^. Human FSGS-causing mutations in anillin have been shown to induce hyperactivation of both Rac1 and mTOR in podocytes ^36^. Rac1 activity is also regulated via the kindlin-2–RhoGDIα–Rac1 signalling axis. Knockout of kindlin-2 resulted in hyperactivation of Rac1 via a reduction in RhoGDIα levels and an increased dissociation of this protein from Rac1, leading to podocyte cytoskeletal re-organisation, foot process effacement and massive proteinuria ^37^. In addition, podocyte foot process effacement due to loss of kindlin-2 has been linked to increased RhoA activity and resulting changes to cortical actin structures, plasma membrane tension and focal adhesion function ^38^. Rac1 activity is also regulated by the binding of RhoGDI to the actin regulatory protein, ezrin. The ezrin knockout mouse has significantly reduced Rac1 activity and is protected from injury-induced morphological changes ^39^. CLIC5A, a chloride intracellular channel, stimulates podocyte Rac1 activity leading to the activation of both ezrin and the cytoskeleton regulator PAK1 ^40^.

**Figure 1.**
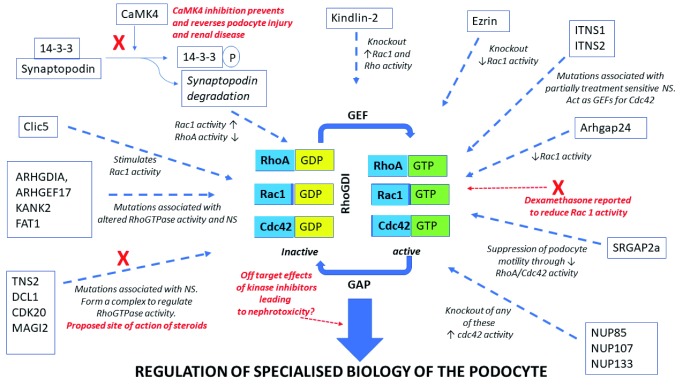
Regulation of the RhoGTPases in the podocyte.

These results suggest that clearly understanding the cellular network in health and disease that both regulate and are regulated by RhoGTPase activity will provide new therapeutic targets for NS. Indeed, small-molecule inhibitors targeting the RhoGTPases are already being developed in the cancer field and several drug targets have been suggested in the NS field, such as TRPC5, TRPC6 and suPAR, which either are known modulators of or are modulated by the RhoGTPases ^16– 19, 41^. For example, inhibition of TRPC5 which is activated downstream of Rac1, leading to deleterious podocyte cytoskeleton remodelling, has been shown to ameliorate kidney disease in rat models of NS ^19^. However, as detailed above, there is already evidence for the involvement of multiple interactions and pathways in maintaining the correct balance of activity of the RhoGTPases and these proteins work in concert with other GTPases such as dynamin to regulate podocyte phenotype ^42^, so unpicking this complex network will not be an easy proposition ( Figure 1).
